# Mitochondrial‐derived vesicles: Recent insights

**DOI:** 10.1111/jcmm.17391

**Published:** 2022-05-18

**Authors:** Lucia‐Doina Popov

**Affiliations:** ^1^ “Nicolae Simionescu” Institute of Cellular Biology and Pathology of the Romanian Academy Bucharest Romania

**Keywords:** extracellular vesicles, lysosomes, Parkin, peroxisome, PINK1, Quality control

## Abstract

The generation of vesicles is a constitutive attribute of mitochondria inherited from bacterial ancestors. The physiological conditions and mild oxidative stress promote oxidation and dysfunction of certain proteins and lipids within the mitochondrial membranes; these constituents are subsequently packed as small mitochondrial‐derived vesicles (MDVs) (70–150 nm in diameter) and are transported intracellularly to lysosomes and peroxisomes to be degraded. In this way, MDVs remove the damaged mitochondrial components, preserve mitochondrial structural and functional integrity and restore homeostasis. An outline of the current knowledge on MDVs seems to be necessary for understanding the potential impact of this research area in cellular (patho)physiology. The present synopsis is an attempt towards the accomplishment of this demand, highlighting also the still unclear issues related to MDVs. Here, we discuss (i) MDVs budding and generation (molecules and mechanisms), (ii) the distinct cargoes packed and transported by MDVs, (iii) the MDVs trafficking pathways and (iv) the biological role of MDVs, from quality controllers to the involvement in organellar crosstalk, mitochondrial antigen presentation and peroxisome *de novo* biogenesis. These complex roles uncover also mitochondria integration into the cellular environment. As the therapeutic exploitation of MDVs is currently limited, future insights into MDVs cell biology are expected to direct to novel diagnostic tools and treatments.

## 1 INTRODUCTION

Mitochondria are essential intracellular organelles well known for their function in generating the energy required to maintain normal cellular processes and safeguard cell homeostasis. Mitochondria execute and coordinate a wide range of metabolic pathways (phospholipid transfer, inflammation, calcium balance, ion homeostasis, aldehyde metabolism, etc.) and contribute to the cell's survival or death. Dysregulated homeostasis implies the damage and dysfunction of mitochondria triggered by various stressors, mainly the reactive oxygen species (ROS), the side‐products of adenosine triphosphate (ATP) biosynthesis. Therefore, the maintenance of a healthy mitochondrial population imposes the clearance of dysfunctional/damaged organelles. In eukaryotic cells, this task is accomplished by the ‘quality control’ (QC) system that engages specific regulatory pathways, depending on the nature and severity of mitochondrial dysfunction.^1^, ^2^, ^3^, ^4^, ^5^, ^6^, ^7^, ^8^, ^9^, ^10^ Examples are (i) the antioxidant enzymes (that cope against ROS‐mediated toxicity), (ii) the activities of the ubiquitin‐proteasome system (that execute the QC of outer mitochondrial membrane, OMM, proteins), and of the mitochondrial proteases, and chaperones (that refold or degrade the damaged/mislocalized mitochondrial proteins), (iii) the packaging of oxidized proteins or lipids (resulting from exposure to subtoxic doses of stressors such as glucose oxidase and xanthine oxidase/xanthine) into mitochondrial‐derived vesicles (MDVs); these vesicles are subsequently delivered to the late endosomes/lysosomes or to the peroxisome for degradation^1^, ^8^, ^10^ and (iv) the autophagic elimination (mitophagy) of extensively damaged mitochondria, in response to stressors such as the strong oxidants.^4^ Compared with the latter pathway, MDVs allow degradation of not yet depolarized mitochondria, are a kinetically faster QC route, and help the clearance of defective mitochondrial proteins and lipids, before executing the degradation of the entire organelle by mitophagy.^1^, ^10^, ^11^ The failure of the QC systems above intervenes in human neurodegenerative diseases and cardiomyopathy.^8^, ^12^, ^13^ For reviews, see.^14^, ^15^, ^16^

The ability of mitochondria to load their contents into vesicles is a constitutive and evolutionary conserved process, inherited from the bacterial ancestors.^16^, ^17^ The bacterial vesicles are generated by the local expansion of the envelopes, the budding of the vesicles and their release extracellularly. Although a large diversity of bacterial envelopes exist, two main types of extracellular vesicles (EV) are prevalent: the outer membrane vesicles (OMVs) derived from the outer layer, and the outer‐inner membrane vesicles (O‐IMVs) containing both outer and inner constituents.^18^ The composition of OMVs includes the outer layer lipopolysaccharides (LPS), peptidoglycans of the periplasmic space, and cytosol.^19^ The O‐IMVs include DNA, cytoplasmic and inner membrane proteins, and ATP.^20^, ^21^ The biological role of bacterial EV consists in signalling within the colony *via quorum sensing*,^22^ intercellular communication by transport of proteins, modulation of immunogenic host invasion, execution of bacterial competitors and biofilms formation.^23^ The vesicular transport has been conserved during evolution, and MDVs are an example of this inheritance.

The bacterial EV and MDVs share several elements in common: (i) the single‐/double‐membrane, (ii) the propensity for transport tasks, (iii) the involvement in immune response and (iv) the possibility of EV formation under specific stress conditions.^11^, ^24^, ^25^ Among the differences between the bacterial EV and MDVs one can quote: (i) the peculiar composition of their membrane (see above for bacterial EV, and of mitochondrial origin for MDVs), (ii) the size of OMVs (diameter is up to 500 nm) is larger than that of MDVs, (iii) the particular transport pathways, intercellular, for the bacterial vesicles and intracellular, for the MDVs engaged in inter‐organellar communication.^1^, ^16^

At present, MDVs define an emerging research area, validated by the recent discoveries on their mechanistic and roles. However, numerous open questions still deserve deeper insights. Therefore, an overview of the literature is necessary to bring the unsolved issues to attention and focus on the MDVs potential exploitation in therapy. The following subjects are discussed here: (i) MDVs budding and generation (molecules and mechanisms), (ii) the identity of cargoes packed and transported by MDVs, (iii) the MDVs trafficking pathways and (iv) the biological role of MDVs. The review is concluded by (v) the future perspectives of this dynamic research area.

## 2 MDVs BUDDING AND FORMATION (MOLECULES AND MECHANISMS)

The MDVs have a small size (diameter between 70 and 150 nm), as observed by electron microscopy; these vesicles are generated by mitochondria in both basal and stress‐related conditions, independent of the fission protein, dynamin‐related protein 1 (Drp1).^1^, ^3^, ^8^, ^11^, ^26^ Several molecules intervene in the budding of damaged segments of mitochondria into MDVs; vital are the Parkinson's disease‐associated proteins PINK1, Parkin and the Vacuolar sorting protein 35 (Vps35).^10^, ^27^, ^28^, ^29^ PINK1 (PTEN‐induced kinase 1) is a mitochondrial serine/threonine‐protein kinase (encoded by the *PINK1* gene), and Parkin is an E3 ubiquitin protein ligase; in terms of structure, Parkin contains at its N terminus a ubiquitin‐like domain (Ubl) and four zinc‐coordinating RING‐like domains. The Parkin‐dependent ubiquitination of mitochondrial proteins is the central mechanism involved in the elimination of the damaged segments of mitochondria.^27^

In mildly oxidative stress conditions, the proteins of the mitochondrial membranes become oxidized; moreover, ROS and oxidative stress initiate the local activation of PINK1 and Parkin, leading to the budding of oxidized membrane proteins into vesicles.^10^, ^30^ As observed in Parkinson's disease, the loss of PINK1/Parkin‐mediated MDVs formation is due to the inability of mitochondria to remove the oxidized/ damaged proteins, leading to mitochondrial dysfunction.^10^ The role of Vps35 in MDV generation is acknowledged by the fact that its mutation impairs MDVs formation.^28^

The restoration of mitochondrial homeostasis by the removal of oxidized proteins *via* MDVs is upregulated in stress conditions,^8^ under remote ischaemic preconditioning,^31^ and in connection with cannabidiol treatment.^32^ This compound (C_21_H_30_O_2_) is the major non‐psychoactive phytocannabinoid, used in nutraceutical and medical treatment. Recently, Ramirez et al.^32^ showed that cannabidiol generates MDVs *via* the PINK1‐Parkin pathway, and heals dysfunctional mitochondria by opening the mitochondrial permeability transition pore. According to the working hypothesis of PINK1/Parkin‐mediated MDVs formation,^27^ the mechanism implies four steps: (i) ROS and/or defects in protein assembly direct the aggregation of oxidized or unfolded proteins within the matrix; in this stage, cardiolipin oxidation generates phosphatidic acid, a contributor to the alteration of membrane curvature, (ii) the protein aggregates saturate chaperones, affecting the import of ‘an individual’ channel in a process affected by cardiolipin oxidation; PINK1 is imported fast and accumulates at the site of the failed import channels, (iii) next, PINK1 phosphorylates ubiquitin and the ubiquitin‐like domain of Parkin, an event with two consequences: it stabilizes activated Parkin and facilitates MDVs generation; (iv) MDVs are formed and released. Matheoud et al.^30^ showed that MDVs biogenesis requires the recruitment of Rab9 (a small GTPase associated with pathways towards the endo‐lysosomal compartments) and of Sorting nexin 9 (SNX9), although the regulatory process is still incompletely understood.^26^

Among the techniques currently used for MDVs evaluation, one can quote the transmission electron microscopy, flow cytometry, electron tomography analysis, immunofluorescence and confocal microscopy.^1^, ^31^, ^33^, ^34^

## 3 THE IDENTITY OF CARGOES PACKED AND TRANSPORTED BY MDVs

The mild oxidative stress induces the packaging of mitochondrial oxidized proteins into single or double‐membrane vesicles; these will be ultimately targeted to lysosomes for degradation.^11^, ^14^, ^32^ Specific mitochondrial compartments are employed for vesicles generation. Thus, of the OMM, the single‐membrane MDVs recruit all ß‐Barrel proteins,^12^ the mitochondrial‐anchored protein ligase (MAPL or Mul1),^28^ and the translocase of the outer membrane 20 (TOM20).^35^ The TOM complex is evaluated to be ‘the entry gate’ for the precursor proteins biosynthesized on cytosolic ribosomes.^36^, ^37^ The double‐membrane MDVs are generated from both OMM and inner mitochondrial membrane (IMM) carrying sometimes matrix proteins.^3^, ^8^, ^10^, ^11^, ^15^, ^27^ Of the IMM, MDVs incorporate specifically the OXPHOS complexes, complexes III and V, and the Fe‐S cluster; the latter functions in preventing mitochondrial Fe overload and in the removal of the irreversibly damaged proteins.^17^ Of the mitochondrial matrix, MDVs might include pyruvate dehydrogenase,^38^ the TCA cycle, fatty acids ß‐oxidation^17^ and SOD2.^11^, ^26^ Moreover, mtDNA can be transferred by MDVs, a process associated with systemic inflammation in Parkinson's disease.^39^ In this disorder, the defective complex I activity is due to the mutations in (NADH): ubiquinone oxidoreductase subunit S3 (NDUFS3).^40^ Another group of MDVs transfers both OMM‐related TOM20 and the matrix SOD2.^11^, ^26^

Besides oxidative stress, hypoxia induces MDVs formation. Thus, when loaded with Bcl‐2, MDVs inhibit mitochondrial apoptosis, and help alleviate myocardial ischemia.^34^ In addition, the stress induced by starvation generates also MDVs which are transported to lysosomes for the subsequent degradation.^10^, ^11^

Recently, proteomic analysis acknowledged the presence of 107 high‐confidence cargoes in TOM20 positive MDVs.^12^ In brain MDVs, have been identified 72 proteins (31% from the OXPHOS) along with the small TIM chaperones.^13^, ^41^ In cardiac MDVs, Vasam et al.^17^ reported the occurrence of proteins containing hyper‐reactive cysteine residues, redox enzymes and enzymes that mediate iron metabolism. These examples may imply MDVs tissue specificity.

In living organisms, MDVs are a heterogeneous population of vesicles.^23^, ^42^ Their selectivity in cargo incorporation is commanded by the nature of the mitochondrial stress.^11^, ^27^ According to Ryan and Tumbarello,^42^ the cargo and ‘potentially’ the membrane constituents outline three attributes of MDVs: the trafficking mechanism employed, the intracellular route and their ultimate destination.

## 4 THE MDVs TRAFFICKING PATHWAYS

**FIGURE 1:**
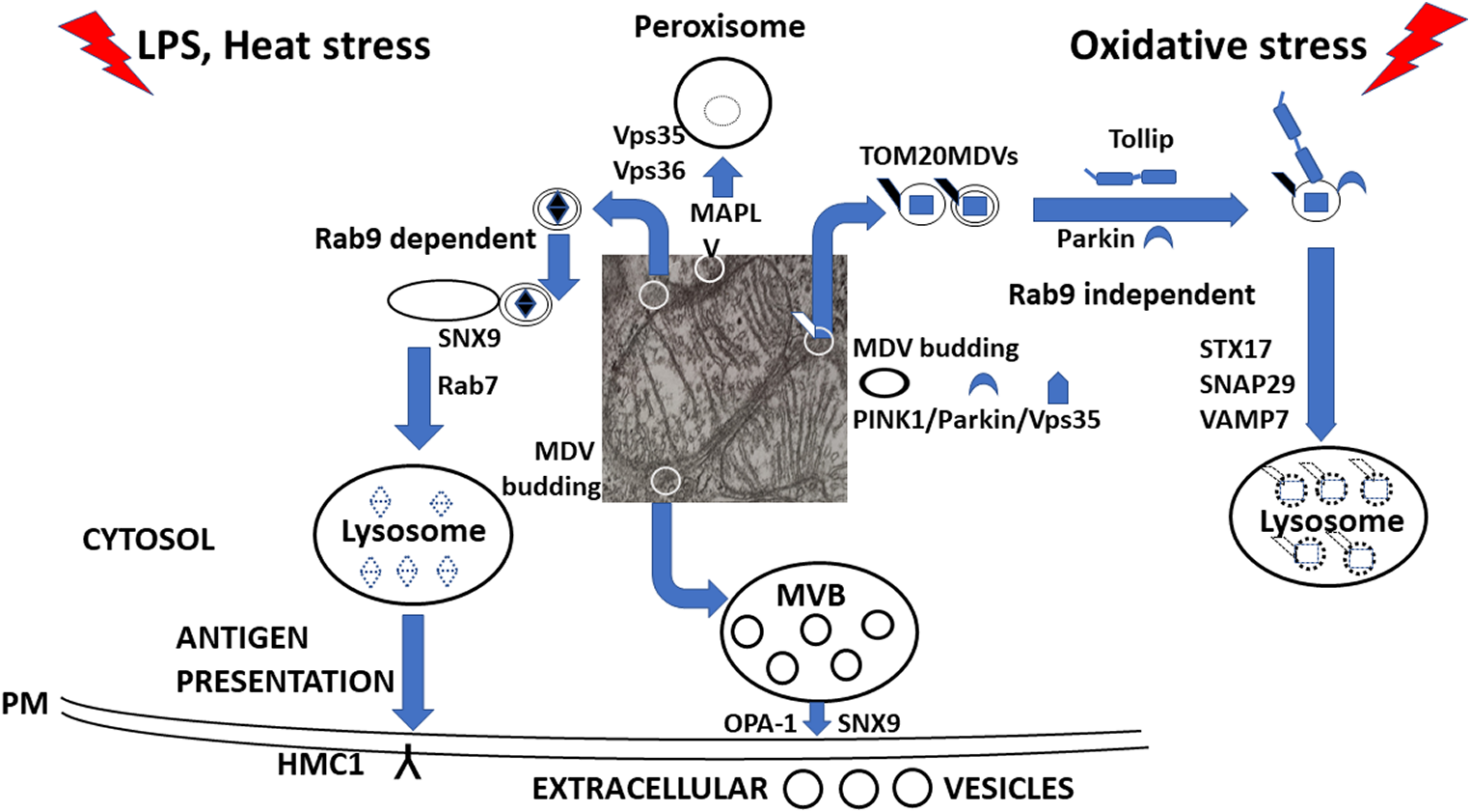
Outline of the MDVs generation and trafficking routes

The specific destinations of MDVs trafficking are the lysosomes, the peroxisomes, the bacterial phagosomes and the extracellular vesicles (EV). The lysosomal route is mainly taken by MDVs containing oxidized proteins and engages the PINK1/Parkin pathway^10^, ^11^, ^27^, ^41^ (Figure 1). The loss of this pathway limits the mitochondria's capability to degrade the damaged proteins, followed by mitochondrial dysfunction. Such a defective process occurs in Parkinson's disease^10^ and is due to the mutations of the corresponding genes. Recent reports demonstrate that the recessive early‐onset Parkinson's disease is associated with biallelic mutations in PINK1^43^ and the loss‐of‐function mutations in the *PARK2* gene, resulting in Parkin depletion.^44^ In Parkin‐deficient mice, mutations in the mouse Parkin gene (Park2) are accomplished by the targeted deletion of Parkin exon 2.^45^

Recent studies uncovered both the proteins beyond the MDVs flux to lysosomes/endo‐lysosomal compartment and those required for the fusion between the MDVs (containing oxidized proteins and lipids) and the degradative organelle. Thus, in response to mitochondrial stress, the endosomal adaptor Toll interacting protein (Tollip), in coordination with Parkin enables the transport of single‐membrane TOM20 MDVs to the endo‐lysosomal compartment,^35^, ^42^ following a Rab9 GTPase independent pathway.^10^ Subsequently, MDVs fusion with the lysosomes is facilitated by the SNARE protein Syntaxin‐17 (STX17) that forms a complex with SNAP29 and VAMP7; this process is Rab9‐independent.^10^ Next, a retromer‐dependent pathway could allow the ‘recycling’ of Tollip back from lysosomes to the early endosomes, either directly or via Rab7.^35^

Another group of MDVs targets the peroxisome^6^ (Figure 1). MDVs fusion with peroxisome, followed by the delivery into the peroxisome lumen is still unclear. However, a small number of contributing proteins have been identified so far.^14^ Thus, MAPL‐containing MDVs are transported to the peroxisome,^27^, ^28^ and the recruitment involves two components of the retromer complex: Vps35 and the Vacuolar sorting protein 36 (Vps36). This complex is known for regulating the vesicle's retrograde transport from the endosomes to the trans‐Golgi network.^28^

The MDVs could also converge to the bacteria‐containing phagosomes. Abuaita et al.^26^ demonstrated that the infection of macrophages with methicillin‐resistant *Staphylococcus aureus* stimulated the generation of MDVs packed with SOD2. The loaded MDVs are delivered to the bacteria‐containing phagosomes, where SOD2 converts superoxide anions ($O_{2}^{‐\cdot }$) into hydrogen peroxide (H_2_O_2_), which is used to kill the invading bacteria; this is an example of MDVs operation in antimicrobial defense.^34^ One can conclude that MDVs trafficking pathways (described above) prove the interaction of MDVs with certain intracellular organelles.

Under stress conditions, the lysosomal degradation may be exceeded, and the MDVs containing dysfunctional parts could operate as pro‐inflammatory damage‐associated molecular patterns (DAMPs); cells prevent such an event by packaging the MDVs inside EV that will be extracellularly discharged^17^, ^34^, ^46^ (Figure 1). Recently, Todkar et al.^46^reported that the EV route is followed by MDVs containing two proteins: the optic atrophy 1 (OPA1) and the SNX9. Moreover, the presence of Parkin inhibits this route, and MDVs are targeted for lysosomal degradation.

## 5 BIOLOGICAL ROLES OF MDVs

There are several generally acknowledged roles of MDVs:

- a player in mitochondrial QC, ensuring the preservation of mitochondrial proteome (containing >1,000 proteins) and the functional integration of mitochondria according to the cellular demands.^8^, ^15^, ^17^, ^26^, ^34^, ^38^ Recently, Lv et al.^31^ affirmed that MDVs act as ‘the first line of defence’ for the removal of the harmed mitochondrial components, before the degradation of the entire organelle by mitophagy. In cells where mitophagy is inactive or defective (such as the cancer cells), the MDVs pathway operates as a compensatory, adaptive mechanism supporting mitochondrial health.^33^, ^47^, ^48^ The potential therapeutic targeting of mitochondrial QC pathways is particularly important for the alleviation of mitochondrial dysfunction in pathophysiology^13^, ^49^, ^50^, ^51^;
- MDVs maintain the mitochondrial turnover, as the degraded damaged cargo could be replaced by novel proteins and lipids *via* biogenesis^52^;
- MDVs participate in inter‐organellar communication, an additional evolutionary conserved trait of mitochondria.^3^, ^16^, ^17^, ^26^, ^34^ While transported within the cytosol, MDVs routes converge to the endo‐lysosomal compartment, to the lysosomes, and to the peroxisome and exchange proteins and lipids at the contact sites; moreover, in hypoxic conditions, mitochondria enriched in Bcl‐2 could transfer it to less healthy mitochondria^34^;
- MDVs mediate mitochondrial antigen presentation (MitAP), a process important in immune tolerance and immune responses.^30^ These vesicles are generated in response to LPS exposure or heat stress. Their formation requires the presence of Rab7 (a small GTPase that monitors vesicular transport to late endosomes and lysosomes), Rab9, and SNX9, and is inhibited by PINK1 and Parkin.^17^, ^30^, ^34^ This set of MDVs is transported also to the lysosomes where the mitochondrial antigens are processed, and MHC class I molecules are presented at the cell surface^30^ (Figure 1);
- a distinct group of MDVs are implied in *de novo* peroxisome biogenesis^2^, ^53^ and are not subjected to lysosomal degradation.^53^ Previously, it was demonstrated that MDVs containing the E3 ubiquitin ligase MAPL target peroxisome.^27^ This small organelle is born either by the growth and division of the existing cellular population or by *de novo* biogenesis.^54^ Although the latter issue was extensively documented in yeasts, fewer studies deciphered it in mammaliancells.^55^ According to the recent results, immature pre‐peroxisomes are formed by the fusion of vesicles containing peroxisome biogenesis‐initiating proteins known as ‘peroxins’ (Pex).^2^, ^54^ Interestingly, the vesicles involved in the fusion originate from two different organelles: the mitochondria, which bud MDVs enriched in Pex3 (descended from OMM)/Pex14 (an integral membrane protein implicated in peroxisomal matrix import), and the endoplasmic reticulum, that generates Pex16 holdng vesicles. The resulting fused structure imports peroxisomal membrane proteins into the lipid bilayer (with the contribution of Pex3 and Pex16), and recruits the matrix (lumen) proteins from the cytosol.^54^ The fully competent mature peroxisome continues these imports, grows, elongates and divides into two to five ‘daughter’ organelles, adjusting their abundance, according to the cellular demands.^2^, ^54^ However, numerous questions are still open and deserve further insights. Thus, Schrader and Pellegrini^55^ mention several gaps in the mechanism of OMM Pex3 transport and engagement in MDVs fusion, the details of Pex16 and Pex3/Pex14 vesicles fusion, and the understanding of the maturation process. The peroxisome is a ubiquitous organelle, with numerous cellular functions, including the involvement in ROS and lipid metabolism. The dysregulated activity of peroxisome in certain diseases is another issue worth to be unveiled by further research.
- MDVs could operate in antimicrobial defence^26^ (discussed in section #4).
- MDVs have a broad biomedical significance. This extends from myocardial ischemia^34^ and neurodegenerative diseases^12^, ^27^ to skeletal myocytes,^56^ liver,^50^, ^57^ brown adipose tissue^58^ and cancer cell metabolism,^47^ to give a few examples only. Thus, in skeletal myocytes MDVs contribute to the maintenance of mitochondrial homeostasis, and to the immune signalling associated with muscle remodelling.^56^ Previous reports demonstrated that MDVs have a protective role against alcohol‐induced liver injury.^50^ However, these vesicles are absent or decreased in the liver of Parkin knock‐out mice.^57^ A recent report acknowledges that the release of EV can be considered a biomarker in liver diseases.^59^ In the brown adipose tissue, mitochondrial‐derived EV reduced the PPARγ signalling and the levels of uncoupling protein 1 (UCP1).^58^ In human renal cell carcinomas, vesicles of mitochondrial and endoplasmic reticulum origin have been observed by electron microscopy.^60^ In addition, the rare autophagy‐deficient clones are characterized by MDVs increased levels^47^; this is an adaptation that compensates for autophagy loss and targets mitochondrial homeostasis maintenance.^61^

## 6 FUTURE PERSPECTIVES

The urgent issues to be clarified include the uncovering of regulatory events behind MDVs generation in the cardiovascular diseases pathophysiology,^15^, ^32^ deciphering the relationships between the QC pathways,^10^, ^62^ disclosure of the potential mechanisms beyond coordination of mitochondrial‐lysosomal axis and EV trafficking,^14^ characterization of mitochondrial EV in various pathologies,^8^, ^14^, ^15^ stimulation of cardiomyocytes to generate Bcl‐2 containing MDVs, potentially useful for the therapy of myocardial ischemia^34^ and finding those mitochondrial pathways that could delay inception of neurodegenerative diseases.^12^, ^27^ Besides the above objectives, one should be aware that the present knowledge on MDVs is based on *in vitro* studies; therefore, *in vivo* identification of MDVs metabolism (generation, wrapping and transport to the correct intra(extra)cellular organelle) using animal models is crucial for their potential use in clinical settings.^34^

## AUTHOR CONTRIBUTION

**LUCIA DOINA POPOV:** Conceptualization (lead); Formal analysis (lead); Visualization (lead); Writing – original draft (lead); Writing – review & editing (lead).

## CONFLICT OF INTEREST

The author confirms that there is no conflict of interest.
